# Examining the Self-Harm and Suicide Contagion Effects of the Blue Whale Challenge on YouTube and Twitter: Qualitative Study

**DOI:** 10.2196/15973

**Published:** 2020-06-05

**Authors:** Amro Khasawneh, Kapil Chalil Madathil, Emma Dixon, Pamela Wiśniewski, Heidi Zinzow, Rebecca Roth

**Affiliations:** 1 Department of Anesthesiology and Critical Care Johns Hopkins University Baltimore, MD United States; 2 Glenn Department of Civil Engineering Clemson University Clemson, SC United States; 3 Department of Industrial Engineering Clemson University Clemson, SC United States; 4 College of Information Studies University of Maryland, College Park College Park, MD United States; 5 Department of Computer Science University of Central Florida Orlando, FL United States; 6 Department of Psychology Clemson University Clemson, SC United States

**Keywords:** suicide, suicidal ideation, self-mutilation, mental health, self-injurious behavior, behavioral symptoms

## Abstract

**Background:**

Research suggests that direct exposure to suicidal behaviors and acts of self-harm through social media may increase suicidality through imitation and modeling, particularly in more vulnerable populations. One example of a social media phenomenon that demonstrates how self-harming behavior could potentially be propagated is the blue whale challenge. In this challenge, adolescents and young adults are encouraged to engage in self-harm and eventually kill themselves.

**Objective:**

This paper aimed to investigate the way individuals portray the blue whale challenge on social media, with an emphasis on factors that could pose a risk to vulnerable populations.

**Methods:**

We first used a thematic analysis approach to code 60 publicly posted YouTube videos, 1112 comments on those videos, and 150 Twitter posts that explicitly referenced the blue whale challenge. We then deductively coded the YouTube videos based on the Suicide Prevention Resource Center (SPRC) safe messaging guidelines as a metric for the contagion risk associated with each video.

**Results:**

The thematic analysis revealed that social media users post about the blue whale challenge to raise awareness and discourage participation, express sorrow for the participants, criticize the participants, or describe a relevant experience. The deductive coding of the YouTube videos showed that most of the videos violated at least 50% of the SPRC safe and effective messaging guidelines.

**Conclusions:**

These posts might have the problematic effect of normalizing the blue whale challenge through repeated exposure, modeling, and reinforcement of self-harming and suicidal behaviors, especially among vulnerable populations such as adolescents. More effort is needed to educate social media users and content generators on safe messaging guidelines and factors that encourage versus discourage contagion effects.

## Introduction

### Background

Adolescents and young adults are the largest population using the internet as it has become essential for their schoolwork, information collection, and socializing [1-6]. Communication tools on the internet, including emails, direct texting, and blogging, have become fundamental in adolescents’ social development [7-9]. More recently, web-based social networks, also known as social media, such as Facebook, Instagram, Twitter, and YouTube, have become increasingly popular and common among adolescents as sites where they can develop public accounts or profiles to connect with other individuals and see their list of connections and posts [7,9-11].

Although social media has created a number of opportunities for individuals to garner social support on the web [1,2,12-14], it also has the potential to negatively impact vulnerable individuals. Recent studies have highlighted how social media can be used to harass, discriminate [15,16], dox [17], and socially disenfranchise individuals [18-20]. Some research even suggests that social media use may be a contributing factor to the significant increase in suicide rates and depressive symptoms among adolescents in the past decade [1,21,22]. Evidence suggests that suicidal behavior can be propagated through social contagion effects, which occur when self-harming behavior is modeled, normalized, and reinforced in media depictions [23-25].

The public health and psychological literature has established that nonsuicidal self-injury (NSSI), or the purposeful infliction of damage to one’s body through cutting, burning, or bruising, can be propagated through social modeling or imitating the behaviors of those we observe [23,26]. Similarly, direct exposure to suicidal behaviors through peers and/or media leads to an increase in suicidality through imitation and modeling [27,28]. These effects are referred to as suicide contagion and are most notable in adolescents and young adults [26,29,30]. There is a strong relationship between stories of suicide in traditional media and a subsequent increase in suicide rates [31,32], especially for prominent stories [33]. Vulnerable adolescents with preexisting mental health conditions and suicide risk factors are at a higher risk to perceive maladaptive self-injurious behavior as an effective coping strategy [34], particularly when they see others use these behaviors to achieve an attractive goal such as garnering the attention of others [35].

Depictions of suicide and self-harm in traditional media have been shown to have harmful effects on vulnerable individuals, even when they describe a false or fictional behavior [33,36]. Through social contagion, these harmful behaviors may occur more frequently because of the repetitive exposure and modeling via social media, especially when such content goes *viral* [23-25]. Virality is defined as “achieving a large number of views in a short time period” [37]. Such widespread exposure to harmful or suggestive content is particularly detrimental to vulnerable individuals, including adolescents and young adults [35,38]. As a result of research indicating contagion effects related to medial portrayals of suicide and self-harm behavior, the Suicide Prevention Resource Center (SPRC) developed a list of safe and effective messaging guidelines to advise media outlets on the means for reducing the contagion risks and harmful effects associated with media portrayals of suicidal behavior [39]. However, these guidelines are not consistently applied across media outlets, and they have not been specifically tailored for messaging via social media.

In the social media literature, Facebook’s controversial *emotional contagion* study provides large-scale empirical evidence that indirect interactions via social media can also unknowingly influence one’s emotional state [40-42]. For instance, researchers interviewed 90 inpatient adolescents with a history of NSSI, finding that most of the participants were exposed to NSSI via traditional or social media before engaging in self-harming behaviors [24]. Recent attention has focused on how social media, in particular, may play a role in mental health [43] and suicide risk [44,45]. Social media may influence suicidality via factors such as cyberbullying, peer pressure in forums to engage in self-injurious behavior, and glamorized graphic videos and images depicting lethal means used in such behavior [46,47]. In addition, exposure to certain methods of self-injury was directly related to engaging in this behavior, and the more frequently youth were exposed to self-injury–related content in the media, the more frequently they engaged in self-injurious activities [24]. However, exposure to self-injury alone does not relate to engagement in this behavior; rather, it is the frequency of the exposure to self-injury as a coping mechanism that can result in engagement [24]. The necessity for studying this contagion effect in the era of social media is that it can be spread and propagated through web-based social networks more rapidly and widely than through traditional media [48]. Such widespread exposure to harmful or suggestive content is particularly detrimental to vulnerable populations, including adolescents and young adults [35,37]. Thus, suicide contagion is the theoretical framework that motivates this study.

Most of the literature regarding adolescent web safety focuses on sexual or aggressive behaviors that put youth at risk [49]. Unfortunately, few studies have examined how social media can influence adolescents and young adults to engage in self-harming behavior [24,46,50]. Therefore, interdisciplinary research is beginning to highlight the urgent need to form a cohesive research agenda around digital self-harm [50], web-based NSSI [51], the use of social media to discuss deliberate self-harm acts [52,53], and acts of cybersuicide [52,53]. Most of this work, however, is in its early stages, with few drawing upon the developmental aspect of vulnerable adolescents’ social media use.

### Objective

One example of viral self-harming behavior that has generated significant media attention is the blue whale challenge. Allegedly, in this challenge, adolescents and young adults are encouraged to engage in self-harm and eventually kill themselves. The media claims that this challenge, which was first released in Russia in 2013, includes a series of 50 challenges sent directly to teens, each with increasing levels of self-harm and isolation [54]. According to the media, the creator of the game, Philipp Budeikin, a Russian psychologist, wanted to “cleanse the society of biological waste,” meaning the people who are willing to kill themselves for the game are *biological waste* [55]. Although there is insufficient evidence to support the existence of the challenge, we believe its portrayal on social media (or any similar medium) has the potential to impact vulnerable individuals. Research exploring ethical concerns related to the blue whale challenge, the effects the game may have on adolescents, and potential governmental interventions is needed. However, we are unaware of any research that examines potential suicide and NSSI contagion risk regarding the challenge. To address this gap in the literature, this study uses qualitative analysis research techniques to provide empirical evidence of the risk associated with self-harm and suicide contagion through the portrayal of the blue whale challenge on YouTube and Twitter posts. More specifically, the purpose of this study was to explore the following questions:

*Research question 1:* How is the blue whale challenge presented and described on YouTube and Twitter?

*Research question 2:* To what extent are YouTube videos compliant with the safe and effective suicide messaging guidelines provided by the SPRC?

## Methods

### Study Overview

In this study, we selected 2 social media platforms for data collection, YouTube and Twitter, as these are among the most popular social media platforms used by youth and young adults [56]. We identified the common themes of YouTube videos, comments on those videos, and Twitter posts by conducting a thematic analysis [57] on the data extracted from these platforms. The methodology we followed is an iterative qualitative coding process that starts by reviewing the raw data by multiple researchers and ends with a set of themes that represents the majority of the data [57]. In addition to the thematic analysis, we deductively coded the YouTube videos based on the SPRC safe and effective messaging guidelines to explore if these videos violated or adhered to these guidelines, the extent of violation, and which guidelines were violated more frequently than the others. To protect the identity of the users, we have not included any specific information regarding the user’s names or their web identifier in the paper. In addition, the quotes included in the manuscript have been paraphrased without changing the meaning to make it challenging from a search-ability standpoint. Next, we describe in detail how we collected the data from social media platforms, how we conducted the thematic analysis, and how we coded the YouTube videos based on SPRC guidelines.

### Data Collection

YouTube and Twitter were selected as appropriate sources for data collection for 2 reasons. First, both platforms are ranked among the top 20 most popular social media sites. Second, posts on these platforms are normally open to the public [56,58,59]. The videos were searched using the YouTube search engine with the key phrase *blue whale challenge*. Next, we sorted the results by relevance, an option provided by YouTube that ranks the videos in descending order relative to the keyword queries based on several factors including “how well the title, description, and video content match the query and which videos have driven the most engagement for the query” [60]. Covington et al [61] described the algorithm used by YouTube in detail. We collected information from the first 60 videos on the list, which, combined, have a length of around 12 hours. The process of collecting and coding the data was iterative, an approach suggested by Saunders et al [62], to assure data saturation [63], which indicates that “on the basis of the data that have been collected or analyzed hitherto, further data collection and/or analysis are unnecessary” [62]. We did so by beginning the coding process with 40 videos and continued collecting videos until by the 60th video, wherein no more new codes in the data emerged. Interestingly, only 5 of these 60 videos required age verification. The following information was collected for each video: the link, the number of views, and the first 30 comments sorted by *top comments*, if present, as at this point, data saturation was achieved as found by iteratively collecting and coding data [63]. Top comments are identified by YouTube based on how many users like versus dislike a comment. A total of 1112 comments were collected for coding. Inclusion criteria for the videos were (1) relation to the blue whale challenge and (2) in English, translated into English, or contained English subtitles. Inclusion criteria for the comments were as follows: the comment (1) had to be in English and (2) include words. This data collection strategy was chosen to mimic the typical user behavior search strategy [64-66].

In addition to these YouTube videos, 150 Twitter posts were randomly collected using the social media monitoring and analytics tool Salesforce (Radian6) [66], which is a tool that listens, tracks, and analyzes conversations across different web-based channels based on the provided keywords [67]. Although Radian6 can only analyze posts in 10 different languages and is the most expensive social media monitoring tool, it can analyze almost all types of complex posts including forums/news/media, blogs, microblogs, geography, history, and sentiment [68]. These posts were collected from February 2012 to February 2018 using the keywords and inclusion criteria provided in Table 1. The timeline for collecting the data was chosen to cover the period where it is believed that blue whale challenge was most active [69]. We also conducted a Google Trends analysis with the key words *blue whale challenge* and found that the challenge was searched for most frequently from 2017 to 2018, which includes our search period. We initially collected 100 Twitter posts for analysis and continued to collect and code posts until no new codes emerged, meaning all posts collected reflected the existing codebook, with data saturation [62] occurring at 150 posts.

**Table 1:** Keywords, hashtags, and inclusion criteria for collecting Twitter posts using Radian6.

Variable	Term or criteria
Keywords	“Blue Whale Challenge” “Blue Whale Game” “BWC”
Hashtags	“#BlueWhaleChallenge” “#BlueWhaleGame” “#Blue_Whale_Challenge” “#Blue_Whale_Game” “#BWC”
Inclusion criteria	The post had to be in English and related to the blue whale challenge. No duplicates or “reTweets.”

### Thematic Analysis

We conducted a thematic analysis following the 6 phases recommended by Braun and Clarke [57]. First, the researchers familiarized themselves with the data (phase 1) and read and reread the data to note any initial observations. Then, the researchers started the coding process (phase 2) to identify the subthemes (codes) within the YouTube videos, YouTube comments, and Twitter posts. Due to the different policies, technical affordances, and data types of these platforms [50,58,59], we first analyzed each data type separately and then compared similar findings across platforms.

Initial codes were developed by dividing the top 40 videos, their top 30 related comments, and 100 Twitter posts between the 2 researchers. Each researcher then identified a set of codes on their own describing the data found in the videos, comments, and Twitter posts. The 2 researchers then had a meeting to combine their individual sets of codes into 3 comprehensive sets (1 for each type of data being analyzed). We called the resulting set of codes a codebook. The researchers conducted a pilot analysis on the top 40 YouTube videos, 695 YouTube comments under those videos, and 100 Twitter posts to ensure that the initial codebooks sufficiently summarized the data. Following the completion of the pilot analysis, the codebooks were modified by the both researchers together to more accurately summarize the data.

Once the codebooks were finalized, the full analysis of 60 YouTube videos, 1112 YouTube comments under those videos, and 150 Twitter posts was conducted. The 2 researchers separately coded all the data without collaboration to negate bias. Double-coding was allowed for the YouTube videos but not for the YouTube comments and Twitter posts. After coding was complete, they compared their codes with identify conflicts. When the 2 researchers disagreed, they used a consensus-forming process [70], where they discussed the underlying differences in their interpretation until they agreed which theme best suited the data. The final codebooks and results are provided in Multimedia Appendices 1-3. All coding results were entered into Atlas.ti [71], a qualitative coding software, to conduct axial coding and develop emergent themes across the 3 data types (phase 3). Both researchers worked together to tie the related codes into overarching themes. Then, the researchers reviewed the themes to ensure that they represented the data (phase 4). Following this, the researchers named, defined, and wrote about the themes (phases 5 and 6).

### Coding Based on Safe and Effective Messaging Guidelines

The SPRC developed 9 safe and effective messaging guidelines based on best practices from research to reduce the risk of inducing self-harm or suicide-related contagion in those who view a message (Table 2) [33,39,72]. Either following or violating these guidelines represents a metric for the contagion risk associated with a social media post. Each of the YouTube videos was compared with the 9 SPRC safe messaging guidelines by 2 researchers to determine if it adheres or violates each guideline. The researchers conducted 2 rounds of deductive coding [70] based on these guidelines. In the first round, they coded the first 40 YouTube videos individually. Then, they had a meeting to discuss any discrepancy in their coding strategy. Then, they started the second round of coding, where they coded all the 60 YouTube videos. The interrater reliability measured by Cohen κ was 0.66. If the 2 researchers disagreed on a code, the video was set aside for further consensus coding.

**Table 2:** Suicide Prevention Resource Center’s safe and effective messaging for suicide prevention.

Guideline	Description
Emphasize seeking help and provide information on where to find it	Provide steps for finding mental health treatment. Advise that help is available through local service providers and crisis centers and through the National Suicide Prevention Lifeline (1-800-273-8255).
Emphasize prevention	Highlight that suicide is avoidable and preventable and that there are actions for individuals who have suicidal thoughts to prevent them from acting on those thoughts [73].
List the warning signs as well as the risk and protective factors of suicide	List the warning signs like the ones developed by the American Association of Suicidology. List what could both reduce and increase the risk of suicide; these can be found on pages 35-36 in the National Strategy for Suicide Prevention. Educate people on how to identify a person with self-harming thoughts.
Highlight effective treatments for underlying mental health problems	A total of 46% of people who died by suicide from 2014 to 2016 had a known mental health condition, with 67% of them having a history of treatment for substance abuse. Among them, 46.7% had recently been released from a psychiatric facility [74], a percentage which can be reduced by providing improved access to effective treatments and social support [75].
Avoid glorifying or romanticizing suicide or people who died by suicide	Vulnerable younger adults may relate to the attention given to and sympathy for a person who committed suicide [76].
Avoid normalizing suicide by presenting it as a common event	Suicide ideation is not seen as normal by most people, and they do not consider it an option. However, presenting suicide as a common event may remove this bias [77].
Avoid presenting suicide as an inexplicable act or explain it as a result of stress only	Doing so may encourage identification with the victim as well as ignoring the complexity and preventability of suicide. Presenting suicide as explainable or a result of stress only misleads vulnerable individuals to believe that it is a normal response to common life situations [72,77-80].
Avoid focusing on personal details of people who died by suicide	Vulnerable younger adults may feel they are like the person who committed suicide, eventually leading them to consider taking their lives in the same way [79].
Avoid presenting overly detailed descriptions of suicide victims or methods of suicide	Including pictures and/or descriptions of where and how an individual died by suicide may lead a vulnerable person to imitate the act [79,80].

## Results

### Overview

This research focused on 2 main components: first, exploring the types of messages social media users share about the blue whale challenge via YouTube and Twitter, and second, the violation of SPRC guidelines in YouTube videos. We identified the common themes across YouTube videos, YouTube comments, and Twitter posts using an inductive coding approach. Then, we used a deductive coding approach to explore how many of the YouTube videos violated the SPRC guidelines. We subsequently compared these videos with the number of views and finally looked at which of the guidelines tended to be violated more often than others. In this section, we first present the 4 identified themes, and then, we describe the violation of the SPRC guidelines.

### Common Themes Across Social Media Platforms (Research Question 1)

We identified 4 common themes among all the posts in the 3 data types included in this study. Table 3 presents the themes, the corresponding codes (code definitions can be found in Multimedia Appendices 1-3), and the percentages of posts from each type of data. Below, we define each of the themes and provide examples from the extracted data. Note: We paraphrased illustrative verbatim quotes without changing the meaning for the following reasons: (1) to make it challenging to identify the users who made the post and (2) to fix any grammatical inaccuracy or typographical error that have been made by the user that wrote the post or comment.

**Table 3:** Common themes and corresponding codes among the 3 data types.

Theme	Relevant codes from each codebook	YouTube videos, n (%)	YouTube comments, n (%)	Twitter posts, n (%)
Raising awareness about blue whale challenge and discouraging participation	YouTube videos codebook (purpose of the videos—raise awareness; video topics—facts about the challenge; video topics—recommendations) YouTube comments codebook (criticizing the game; interventions and recommendations) Twitter codebook (awareness; information about the challenge)	50 (83)	315 (28.33)	103 (68.7)
Expressing sorrow for people with mental health issues	YouTube videos codebook (purpose of the videos—remembering the victims; Encouragement—negative) YouTube comments codebook (expressing sorrow; encouraging participants and parents) Twitter codebook (mental health)	28 (47)	123 (11.11)	5 (3.3)
Criticizing or making jokes about the participants or the challenge	YouTube videos codebook (purpose of the video—sarcastic, funny, or prank) YouTube comments codebook (criticizing the game; criticizing the victims; sarcastic, funny, or prank) Twitter codebook (sarcastic/funny/jokes)	6 (10)	530 (47.66)	24 (16.0)
Providing experiences and asking to play	YouTube videos codebook (video content—victims related) YouTube comments codebook (participating; personal experience) Twitter codebook (personal experience)	36 (60)	178 (16.01)	1 (0.7)

#### Theme 1: Raising Awareness About the Blue Whale Challenge and Discouraging Participation

This theme included social media posts in which users were trying to raise awareness and warn parents about this dangerous phenomenon. YouTube videos in this context were either news reports or bloggers that started their videos by listing different names for the blue whale challenge and the statistics on how many people died by suicide due to this game. These videos, then, started discussing the tasks involved in the blue whale challenge, including that the only way out of the game is suicide. These videos frequently included clips from interviews with victims’ parents or pictures of the victims while describing the blue whale challenge.

The YouTube comments in the awareness theme were against blue whale challenge and suggested that parents and authorities pay more attention to their children’s safety. For example, 1 of the comments that falls in this category is:

This is getting ridiculous. The government, the Federal Bureau of Investigation, or someone needs to do something about it.

The Twitter posts in this theme centered on awareness of this dangerous game. Users were trying to spread the word to make others aware of and read more about the challenge. One example of the Twitter posts in this theme is:

Please understand and beware of the Blue Whale Challenge. This is for the parents.

This theme was most common in YouTube videos (approximately 83%) and Twitter posts (approximately 68.7%), suggesting that the majority of users on these 2 platforms are trying to spread the word about the danger of blue whale challenge.

#### Theme 2: Expressing Sorrow for People With Mental Health Issues

This theme included social media posts in which the users expressed sorrow for those who participated in the blue whale challenge or people with mental illness issues. These videos primarily presented pictures of these people, accompanied by sad music. YouTube comments in the expressing sorrow theme included words of encouragement and support for people with mental health issues. An example of these supportive comments is:

Love you girl. So many people care about you. Depression is so horrible. Stay strong girl. We love you.

The Twitter posts in the expressing sorrow theme primarily mentioned that people participate in the blue whale challenge because they have mental health issues, urge of self-harm, and suicidal ideation. One example of the Twitter posts reflecting this theme is:

The Blue Whale Challenge: Hey Mr. Scribe, Brush Up Your Archaic Knowledge of Mental Health.

A high percentage of YouTube videos (approximately 47%) fell under this theme, but fewer YouTube comments (approximately 11.10%) and Twitter posts (approximately 3.3%) did.

#### Theme 3: Criticizing or Making Jokes About the Participants or the Challenge

The third theme included social media posts from individuals who either criticized the individuals who participated in the blue whale challenge or made sarcastic comments about them or the challenge. The 6 YouTube videos that fell under this theme (10%) mentioned that adolescents participate in blue whale challenge just to “show off” and criticized them harshly or made sarcastic comments about them. The 511 YouTube comments in this theme (47.70%) criticized the blue whale challenge participants by saying things such as:

People who play this game are more stupid than the game itself. How can someone lose his sense and be manipulated by others like that. Grow up guys. You have brains to think and decide what is good and what is bad for you.

The 24 Twitter posts in the criticizing theme (16.0%) were primarily sarcastic posts about the challenge itself rather than the people who participated in it. For example, 1 of these Twitter posts is:

Husband silently downloaded the Blue Whale Game on his wife's phone. Fifty days later the blue whale committed suicide.

As seen in Table 3, this theme was mainly dominant in the YouTube comments, with a few YouTube videos and Twitter posts falling under this theme.

#### Theme 4: Providing Experiences and Asking to Play

The last theme we identified included social media posts where users spoke in detail about someone who had participated or asked to play the game themselves. The providing experiences theme was very common in the YouTube videos (60%), where these 36 videos interviewed parents and provided pictures of the participants’ bodies showing instances of self-harm. This theme was slightly less common among the YouTube comments (16.00%), and these 178 comments were mainly about experiences of acquaintances or users who were asking to participate in the blue whale challenge. An example of the comments by someone asking to participate in blue whale challenge is:

I want to play the Blue Whale Game. Please give me the link.

Only 2 Twitter posts fell in the providing experience theme (approximately 0.7%), which tended to be acquaintance stories. For example, 1 user posted the following on Twitter:

We just had a meeting here at work and this lady told us that her 10-year-old niece committed suicide because of this Blue Whale Challenge.

A large number of YouTube videos fall under this theme, as opposed to the anecdotal YouTube comments and Twitter posts.

### Safe and Effective Messaging Guidelines (Research Question 2)

Of the 60 YouTube videos evaluated based on the SPRC safe messaging guidelines, 22 (37%) adhered to fewer than 3 of the 9 safe messaging guidelines, meaning that the videos were considered more unsafe than safe. Approximately 50% (30 videos) were considered neutral, meaning they adhered to only 4-6 of the guidelines, whereas the remaining 13% (8 videos) were considered more safe than unsafe because they adhered to 7 or more of the guidelines.

When compared with the number for views of each video, 50% (10 videos) of the top 20 viewed videos were more unsafe than safe, meaning that the videos violated at least 6 of the 9 criteria for safe and effective messaging about suicide. Only 10% (2 videos) of these 20 videos were considered more safe than unsafe, and 40% (8 videos) were considered neutral. The top 20 viewed videos had 46,099,923 views in total. Of the middle 20 most viewed videos, 30% (18 videos) were considered more unsafe than safe, 25% (15 videos) more safe than unsafe, and 45% (27 videos) neutral. The middle 20 viewed videos had 1,169,054 views in total. Of the 20 least viewed videos, 30% (18 videos) were considered more unsafe than safe, 5% (3 videos) more safe than unsafe, and 65% (39 videos) neutral. The 20 least viewed videos had only 123,450 views. All videos met at least one of the nine guidelines, and only 1 video met all 9 criteria. The total number of views for all 60 videos was 47,392,427, with the top 20 videos having 97.27% of the total views.

To better understand which guidelines were most frequently violated, we included the number of videos that violated each guideline in Table 4. The guidelines most frequently violated were “Highlight effective treatments for underlying mental health problems” and “Emphasize seeking help and provide information on where to find it.” As seen in Table 3, most YouTube videos were intended to raise awareness and warn parents and society about the blue whale challenge. It is expected and advisable for these types of videos to provide steps on how to treat such problems or information on where these details could be found, such as the National Suicide Prevention Lifeline [74]. However, as our results indicate, these videos failed to do so.

In addition, most of the videos violated the guidelines “Avoid presenting overly detailed descriptions of suicide victims or methods of suicide,” “Avoid glorifying or romanticizing suicide or people who died by suicide,” and “Avoid focusing on personal details of people who died by suicide.” These videos tended to include personal pictures of the victims, such as pictures of an arm with a whale carved on it. Most of the videos also mentioned that Philipp Budeikin, a 22-year-old dropout psychology student, is the curator of the challenge. They included pictures and quotes from the curator of the game, the most frequently occurring quote as:

The people who have died by the blue whale challenge are biological waste, and I was cleaning the society from them.

A smaller but relatively high percentage of the videos included clips from interviews with the victims’ families and mock conversations between a victim and the curator showing how a person can participate in the challenge. Additionally, they mentioned that the challenge has other names, including but not limited to, *The Silent House, A Sea of Whales, Wake Me Up at 4:20 AM,* and *F57*. The majority of the videos described and provided details about the tasks and methods for attempting suicide in the challenge, including jumping off a high building, jumping in front of a train, and self-hanging. These videos also mentioned specific names of suicide cultures and web-based suicide groups or forums on social media that contributed to the spread of this challenge.

Approximately 67% (40 videos) of these videos violated the guideline “Avoid normalizing suicide by presenting it as a common event.” In these videos, general facts and statistics about the blue whale challenge, such as the number of suicides related to the challenge and the countries in which they occurred were provided. On the other hand, only a few videos provided support hotlines and recommendations to adolescents and parents about how to avoid this game.

**Table 4:** Percentage of videos violating each Suicide Prevention Resource Center safe messaging guideline.

Guideline	Count, n (%)
Highlight effective treatments for underlying mental health problems	54 (90)
Emphasize seeking help and provide information on where to find it	47 (78)
Avoid presenting overly detailed descriptions of suicide victims or methods of suicide	45 (75)
List the warning signs as well as the risk and protective factors of suicide	43 (72)
Avoid normalizing suicide by presenting it as a common event	40 (67)
Avoid glorifying or romanticizing suicide or people who died by suicide	36 (55)
Emphasize prevention	30 (50)
Avoid focusing on personal details of people who died by suicide	30 (50)
Avoid presenting suicide as an inexplicable act or explain it as a result of stress only	20 (33)

## Discussion

### Overview

To the best of our knowledge, our study is the first to systematically document the quality, portrayal, and reach of the blue whale challenge on social media. In this study, we found that it is easy for adolescents to access almost any post about the blue whale challenge on social media, as only 5 of the YouTube videos blocked minors from viewing the content. We assessed the portrayal of the blue whale challenge on social media by investigating the common themes of the videos and posts and the videos’ adherence to the safe and effective messaging guidelines. We found that social media users post about blue whale challenge to raise awareness and discourage participation, to express sorrow for the participants, to criticize the participants, or to describe a relevant experience. Moreover, we found that the majority of the videos on YouTube violated at least 50% of the SPRC safe and effective messaging guidelines. In this section, we discuss in detail the themes found in the results and the potential consequences of the violated SPRC guidelines. We conclude this section by providing the design implications and recommendations to social media platforms and future directions for this research.

### Common Themes Across Social Media Platforms (Research Question 1)

The raising awareness theme was the most dominant theme and included posts that were meant to raise awareness and warn parents and society about the blue whale challenge. These posts were primarily anti-blue whale challenge, as opposed to pro-blue whale challenge. This finding implies that it is difficult to find information on how to participate in blue whale challenge or prosuicide information in comparison with obtaining antisuicide information on the internet, as found in a previous study [81]. It also implies that it is difficult to find videos from actual participants of the blue whale challenge. This finding could partially be due to the nature of the challenge in that it encourages participants to conduct self-harm secretly.

Another topic highlighted in blue whale challenge–related social media posts was that the users felt sorrow for people who participated in blue whale challenge or people with mental illness (theme expressing sorrow for people with mental health issues). This finding parallels our first 2 implications: it is not easy to find pro-blue whale challenge information on social media and even harder to find posts from actual participants. However, posts of this kind make the viewer think that there are a significant number of others participating in the blue whale challenge. This conclusion could lead them to believe that suicide and self-harm are normal responses to common life situations (eg, stress) rather than outcomes that can be prevented [39,76]. On the other hand, as seen in the criticizing theme, there were many posts in which people either criticized the adolescents who participated in the blue whale challenge or made fun of them, agreeing with the purpose of the blue whale challenge to clean “society of people with mental issues.” When people post critical comments about individuals with mental illness, they contribute to the stigma surrounding mental illness and discourage help-seeking behavior [82].

Finally, the providing experience theme showed that a large number of social media users tend to speak in detail about someone who participated in the blue whale challenge, providing their demographics or interviewing their parents or acquaintances who provide more details about the participant’s personal life. This theme was also found by other researchers who examined traditional media posts about suicide [76]. It is possible that reporting this level of detail might contribute to social modeling and contagion by leading vulnerable adolescents to feel that they are similar to the adolescents who participated in the blue whale challenge, making them more likely to participate or harm themselves [79].

### Safe and Effective Messaging Guidelines (Research Question 2)

We suggest that videos similar to those we examined could contribute to the spread of these risky challenges instead of meeting their intended purpose of raising awareness and decreasing participation. Most posts romanticized people who have died by following this challenge, and younger vulnerable individuals may see those victims as role models, possibly leading them to end their lives in the same way [76,81]. In violation of guidelines, the videos presented statistics about the number of suicides believed to be related to this challenge in a way that made suicide seem common [77]. In addition, the videos presented extensive personal information about the people who had died by suicide while playing the blue whale challenge. They also provided detailed descriptions of the final task, including pictures of self-harm and means of suicide, material that may encourage vulnerable adolescents to consider ending their lives and provide them with methods on how to do so [79,80]. On the other hand, these videos both failed to emphasize prevention by highlighting effective treatments for mental health problems and failed to encourage individuals with mental health problems to seek help as well as providing information on where to find it. For these reasons, we believe that these videos could contribute to the propagation of self-injury and suicidality through social modeling and imitation [23,26-28], which is known in the literature as the suicide contagion [29,30]. Suicide contagion is commonly promoted through traditional media [33,36], and we believe it is more likely to occur when social media is employed to depict self-harming behavior, as the content could easily go *viral* [23-25].

The SPRC safe and effective messaging guidelines are provided to help people working in suicide prevention, mental health promotion, or any other forms of media, which ensure that messages about suicide are safe, positive, and strategic [39]. Thus, these guidelines are applicable for assessing the appropriateness and safety of the content in messages in suicide campaigns and those discussing suicide across a variety of platforms [39,83]. Portrayals of both suicide and self-harm on social media platforms have the ability to increase that behavior in viewers regardless of their self-harm history, making the monitoring of self-harm and suicidal content crucial [84]. As adolescents often look for emotional support from the internet within a plethora of potentially harmful content, it is critical that web-based resources follow these guidelines, as not doing so may contribute to and/or increase the likelihood of someone with suicidal thoughts attempting suicide [39,77-80].

### Implications and Recommendations

Although there are no studies investigating how portrayals of the blue whale challenge can contribute to self-harm contagion, messages on social media may affect an individual’s perception, belief, or behavior regarding self-harm. There are also no studies investigating similar challenges that are said to encourage self-harm and suicidal behavior, such as the momo challenge [85]. However, studies have clearly shown the harmful effects of newspapers, movies, and television portrayals that have led to increased rates of suicide and self-harm after violating safe messaging guidelines [33,86-90]. For example, Cooper et al [87] and Ayers et al [88] reported that suicide admission counts increased coinciding with the release of the Netflix series *13 Reasons Why*, which depicts suicide in a highly graphic and unsafe manner. Suicide contagion has been shown across a variety of entertainment and communication media, and social media has been the focus of recent research interest as a medium for contagion because of the ease with which harmful content can be spread [90-94].

As 2 of the most popular websites and social media platforms, YouTube and Twitter are potentially capable of influencing countless adolescents [2,56,59,66,95,96]. With most of the blue whale challenge-related posts on these platforms found to be potentially harmful to vulnerable populations, our findings suggest that it is urgent to monitor social media posts related to the challenge and similar self-harming challenges. The SPRC should appropriately inform social media users, particularly those with greater influence (eg, celebrities and news anchors) on how to address suicide or self-harm in a safe way to reduce contagion. This could be achieved using an algorithm that detects harmful content in the video before posting and provides recommendations to the user to edit or remove such content [97-100]. Although most YouTube users found the blue whale challenge to be a threat and forwarded a message as a means to warn others or speak out against it, they may unintentionally contribute to self-harm contagion. Therefore, it is critical for social media users to evaluate sources before creating and sharing information. They should also be educated on how to respond to unsafe posts and how to report such posts to social media administrators.

YouTube has the potential to become a powerful positive information dissemination platform with the contribution of mental health professionals and organizations [96,97]. Professionals and organizations can do so by actively participating on YouTube channels by creating and uploading videos that are safe, accurate, and trustworthy. Moreover, although only minimal barriers can realistically be applied to video uploads due to the nature of such platforms, there is a need to develop advanced algorithms and interfaces to highlight information that is safe, accurate, and trustworthy. In addition, integrating approved information available from federal agencies such as SPRC might increase the veracity and safety of information available. Greater effort is needed to educate the community about mental health and factors that could lead to self-harm, in addition to educating users on how to respond to unsafe posts. This could be accomplished by adding resources in suicide prevention campaigns or on social media platforms by providing a link on suicide-related videos to educate the public on how to identify and respond to unsafe videos. In addition, features such as crowdsourcing (obtaining information from internet users), asking users to report inaccurate and misleading information to prevent the spread of misinformation, could be integrated on social media sites.

### Limitations and Future Work

There are several limitations to our study, with many related to the data used. We only studied videos and posts about blue whale challenge that are publicly available. It is possible that more harmful videos could be posted on private pages or personal accounts. In addition, we only used hashtags that included blue whale challenge in them to collect the data. It is possible that there are unofficial hashtags that can be used by individuals at high risk of self-harm, allowing them to find related content without censorship. Additionally, the YouTube videos were selected according to their relevance to the topic based on 1 keyword. Ideally, videos should be randomly selected using several keywords and multiple hashtags. Moreover, our sample may differ from videos sampled at a subsequent time as YouTube is rapidly changing by nature. Furthermore, this study focused on 2 social media platforms, whereas people could be posting about the challenge on other platforms including, but not limited to, Facebook, VKontakte, Snapchat, and Instagram. Our study focused on 1 self-harm and suicide challenge, the blue whale challenge, although there are many other self-harm challenges such as the tide pod challenge and other suicide challenges such as the momo challenge.

Future research could include a comparison of the characteristics of posts about different challenges that exemplify various levels of self-harm to better understand the reasons for their viral spread. Understanding these factors will help build simulation models, such as agent-based models, to visualize the spread of these challenges. In addition, understanding these factors will help inform improved policies and interventions for eliminating the spread of harmful challenges among adolescents, such as those found by Luxton et al [47]. Integrating these policies into simulation models will help to identify the policies that are most effective in reducing these challenges with minimal cost.

### Conclusions

We investigated the characteristics of YouTube and Twitter posts focusing on the blue whale challenge and the characteristics of these posts that make them potentially harmful to vulnerable populations. Through our qualitative analysis, we found that although most videos and Twitter posts attempt to raise awareness about the challenge and inform parents, they may have unintentional harmful effects as most violated the SPRC safe messaging guidelines. We conclude that safe messaging guidelines should be more widely disseminated. Our data show that the majority of posters were not professionals and were likely to violate safe messaging guidelines. More efforts are needed to disseminate and educate the average person on messaging guidelines as well as factors that encourage contagion effects.

### Resources for Seeking Help

If you or a loved one has thoughts of suicide or self-harm, please contact the following resource: Suicide Prevention Hotline: 1-800-273-8255.

## Appendix

Multimedia Appendix 1 Codebook for YouTube videos.

Multimedia Appendix 2 Codebook for YouTube comments.

Multimedia Appendix 3 Codebook for Twitter posts.

## Abbreviations

NSSI: nonsuicidal self-injury
SPRC: Suicide Prevention Resource Center
