# Supplementary material for: Examining the Self-Harm and Suicide Contagion Effects of the Blue Whale Challenge on YouTube and Twitter: Qualitative Study
Source: JMIR Ment Health. 2020 Jun 5;7(6):e15973. doi: 10.2196/15973 (PMC7312265; doi:10.2196/15973)
Supplement: Multimedia Appendix 1 [file mental_v7i6e15973_app1.pdf]

Appendix 1. Codebook for YouTube Videos

Code	Sub-codes: Description	Percentage
Media Type	**Digital Media:** Slideshows, animations, or videos without people	37%
	**Blog or Short Film:** Blog or short film depicting a victim doing various tasks	35%
	**News:** News videos, interviews, and chat show panels	23%
	**Personal Video:** Videos recorded using personal phones or other personal devices	5%
Purpose of the Video: based on what is mentioned in it	**Raise Awareness:** Videos explained the challenge, how teens were convinced to participate, provided information about the tasks, and the targeted populations	83%
	**Sarcastic, Funny, or Prank:** Funny videos about the challenge or the video maker sarcastically pretended to participate in the BWC	10%
	**Remembering the Victims:** Pictured slideshows showing individuals who allegedly died by playing the game	3%
Encouragement	**Neutral:** Videos did not encourage nor discourage participation or there was no speaker throughout the video	47%
	**Negative:** Videos expressed sorrow or discouraged people from participating in the challenge	45%
	**Positive:** Videos encouraged the victims or families of victims	8%
Video Content: media used in the video	**Victims Related:** Included photos before, during, or after self-harm as well as quotes from victims	45%
	**Curator:** Videos containing pictures or quotes from the curator	15%
	**Parents:** Contained quotes from or videos of the parents of victims of the BWC	14%
	**Interaction Between Admin and Victim:** Mock interactions of how individuals are approached to enter the game	11%
Video Topics: topics discussed in the video	**Facts About the Challenge:** Videos discussed how many people have died due to the BWC, who created the BWC, which countries have been affected by it, full descriptions of the tasks, and/or the different names for the game	47%
	**Social Media:** Videos talked about suicide culture or suicide groups online	25%
	**Recommendations:** Mentioned some of the interventions by authorities and provided support hotlines as well as recommendations for individuals concerning the BWC	22%
