# Supplementary material for: Examining the Self-Harm and Suicide Contagion Effects of the Blue Whale Challenge on YouTube and Twitter: Qualitative Study
Source: JMIR Ment Health. 2020 Jun 5;7(6):e15973. doi: 10.2196/15973 (PMC7312265; doi:10.2196/15973)
Supplement: Multimedia Appendix 2 [file mental_v7i6e15973_app2.pdf]

Appendix 2. Codebook for YouTube Comments

Code: Description	Illustrative Quote	Percentage (%)
**Criticizing the Game:** Comments which disagreed with the game, asked people not to participate, or asked for the game to be banned	*"It seems like every day I find a new thing to hate about social media."*	23%
**Sarcastic, Funny, or Prank:** Jokes and sarcastic comments	*"I played this game but I stopped when I was instructed to delete my Minecraft account."*	16%
**Encouraging the Video Maker:** Comments praising the person who posted the video for warning the public about the BWC	*"Nice, good, perfect bro."*	11%
**Participating:** Comments in which users expressed their desire to play the game, agreed with the comments made by the creator of the game, agreed with the game, or asked for links to participate themselves	*"I want to play the blue whale game. Please give me the link."*	9%
**Criticizing the Victims:** Comments berating those who had played or said they would like to play the game. These comments were often condescending and insensitive to posters who expressed symptoms of depression	*"You're the saddest human being if you kill yourself to win a challenge."*	8%
**Expressing Sorrow:** Typically pertained to the victims, the victims' families, or the user's own family and friends	*"My heart started crying watching this."*	8%
**Personal Experience:** Included comments in which users revealed that they had played in the past, were currently playing, or someone they knew was playing the game or had played the game but did or did not survive	*"I already attempted this. I bled and had to go* to the hospital."	7%
**Intervention and Recommendation:** Included a phone number or other information for social support for those affected by the BWC. This code also included encouraging other users to report participation in the BWC to parents or to the authorities	*"Let's do a Get Rich Challenge. We all make each other's bank accounts happy. Once person is chosen, a bunch of people send them money, and it goes on and on. Something like that. Someone more intelligent should come up with a formula of some sort*	6%

Code: Description	Illustrative Quote	Percentage (%)
	that'll work. Let's just send each other money, get rich and help each other grow instead."	
**Criticizing the Video:** Contained negative comments about the video quality, content or creator	*"Someone should report these outrageous videos to the authorities."*	5%
**Other:** Related to the BWC but was not summarized by any of the other codes	*"Secret task? That means that they send you something original or what. I'm just curious."*	4%
**Encouraging Teens or Parents:** The comment praised other teens and encouraged them not to participate or encouraged parents to play a role in their child's safety	*"Love you girl. So many people care about you. Depression is horrible. Stay strong girl."*	3%
