# Supplementary material for: Examining the Self-Harm and Suicide Contagion Effects of the Blue Whale Challenge on YouTube and Twitter: Qualitative Study
Source: JMIR Ment Health. 2020 Jun 5;7(6):e15973. doi: 10.2196/15973 (PMC7312265; doi:10.2196/15973)
Supplement: Multimedia Appendix 3 [file mental_v7i6e15973_app3.pdf]

Appendix 3. Codebook for Twitter Posts

Code: Description	Example	Percentage (%)
**Awareness:** Warnings about the challenge directed toward parents, teens, the government or the police. This code also included posts that asked for the game and smartphones to be banned and warned against social media use	*"Parents, you need to know about the deadly Blue Whale Challenge."*	34%
**Information about the Challenge:** Posts about the targeted population, the curators, victims, and/or an estimated number of deaths	*"Blue Whale Challenge: Creator Budeikin has aides to help him in his absence."*	31%
Sarcastic, Funny, or Jokes: Posts that made fun of the challenge or the people who participated in it	*"Engineer downloads Blue whale game: Sorry! But no professionals allowed. *Uninstalls itself*"*	16%
**Opinion**: Either someone voicing their thoughts about the BWC or calling on others to respond to their comment	*"Apex of misuse of psychology. The blue whale game would have been better if that game maker stressed on social welfare."*	13%
**Mental Health:** Informing others about depression and suicidal thoughts among teens	*"The Blue Whale Challenge: What is the psychology behind it?"*	3%
**Personal Experience:** Posts in which the users expressed either their own experiences with the BWC or the experiences of someone they knew	*"We just had a meeting here at work and this lady told us that her 10-year-old niece committed suicide because of this other Blue Whale Challenge."*	1 %
**Other:** Related to the BWC but did not fit under any of the other codes	*"Understanding BLUE WHALE CHALLENGE can tell us how to deal with TERRORISM."*	1%
**Miscellaneous:** Posts that had nothing with the BWC		1%
